# Supplementary figures and images for: Differences in tail feather growth rate in storm-petrels breeding in the Northern and Southern hemisphere: a ptilochronological approach
Source: PeerJ. 2019 Oct 15;7:e7807. doi: 10.7717/peerj.7807 (PMC6798868; doi:10.7717/peerj.7807)

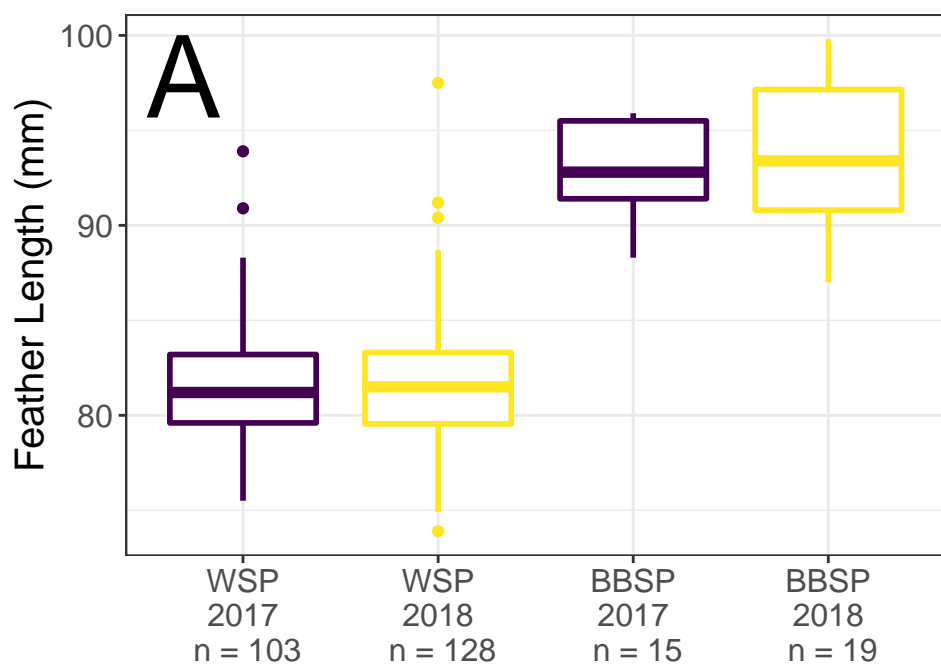

Year 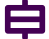 2017 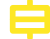 2018

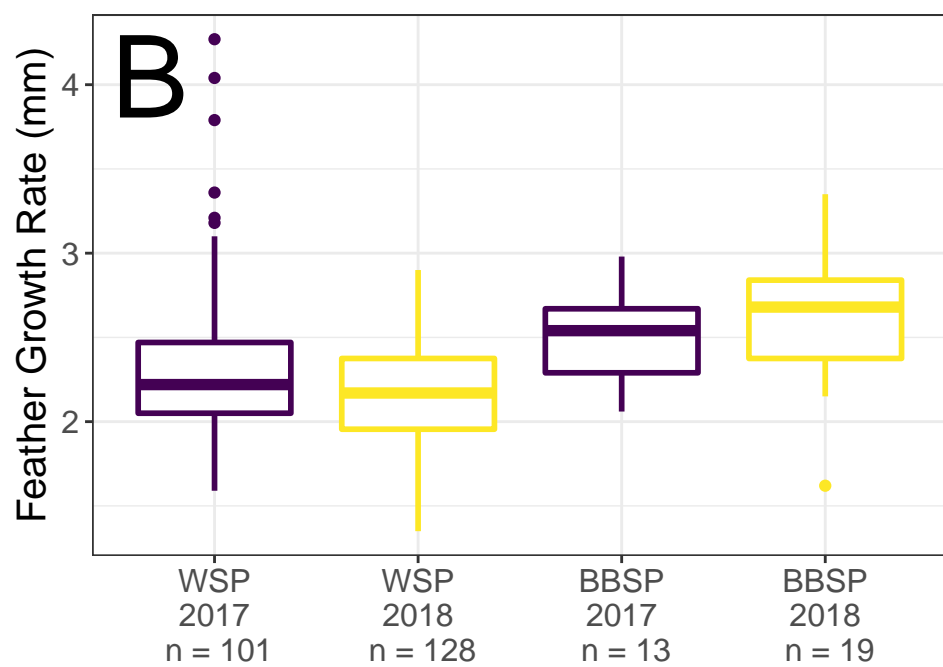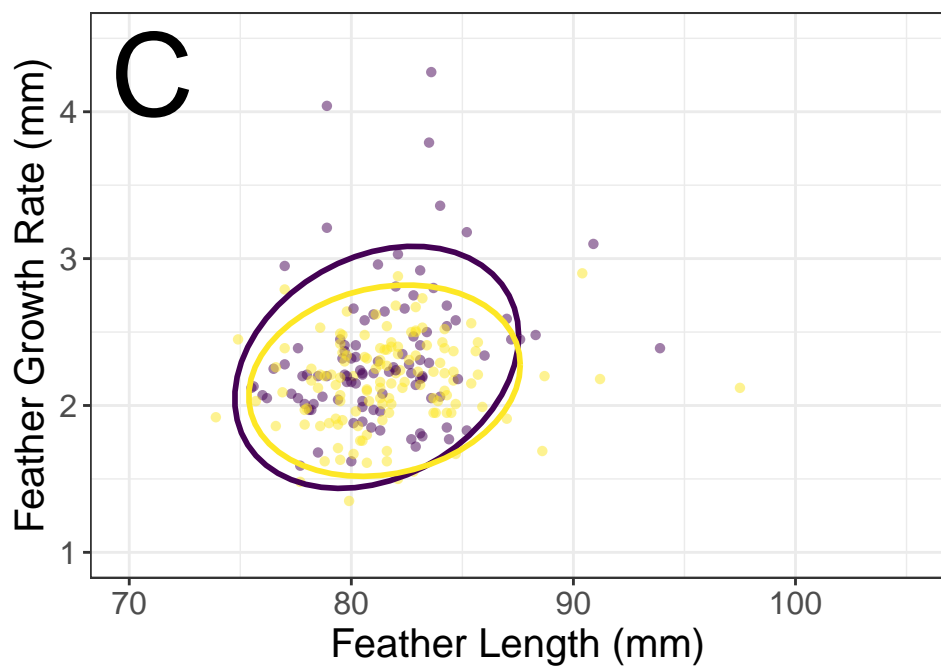

Year 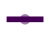 2017 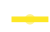 2018

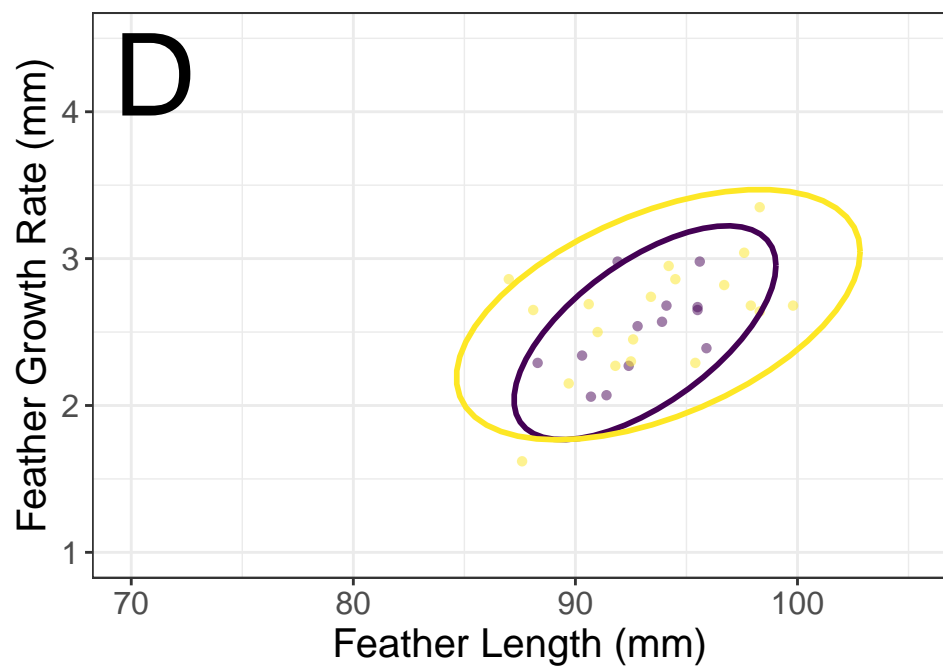

Supplement: Figure S1 — (A) Boxplots showing the inter-annual differences in FL for the Wilson’s (WSP) and black-bellied (BBSP) storm-petrels. 2017 is shown in purple, 2018 in yellow. Student’s t-tests showed significant differences for WSP but not for BBSP (see Materials and Methods), probably because of differences in sample size. (B) Boxplots showing the inter-annual differences in FGR. Student’s t-tests showed significant differences for WSP but not for BBSP (see Materials and Methods), probably because of differences in sample size. (C) Scatterplot showing the overlap between years in FL and FGR for WSP. Ellipses show the 95% confidence level. (D) Scatterplot showing the overlap between years in FL and FGR for BBSP. [file peerj-07-7807-s001.pdf]

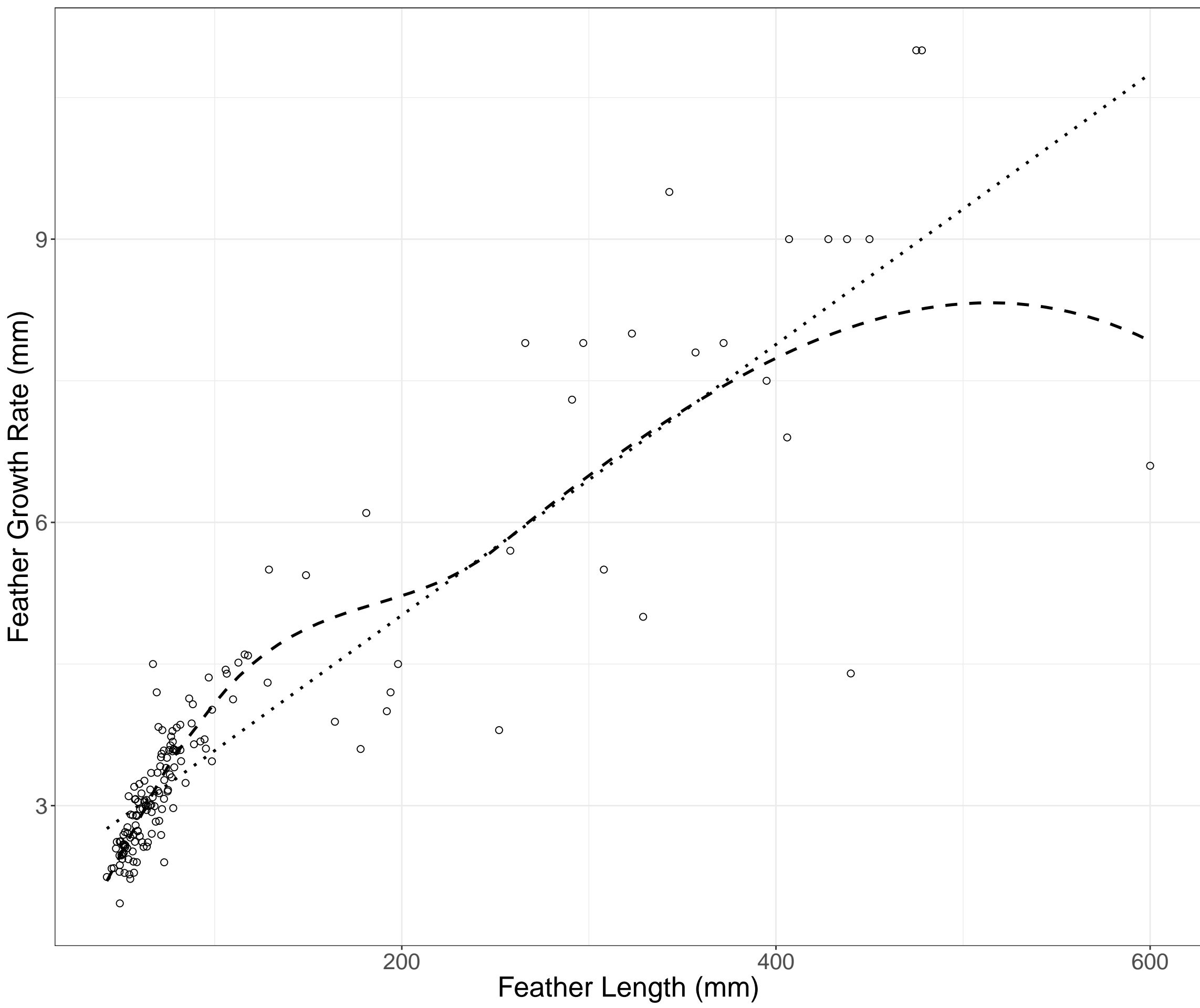

Supplement: Figure S2 — A scatterplot showing the relationship between FGR and FL. Each dot represents the average reported value for a single species (see Table S2, and Materials and Methods for details). The dotted line shows a linear correlation as generated by ggplot (geom_smooth, method = “lm”, package ggplot2) and the dashed line a loess regression. [file peerj-07-7807-s002.pdf]
