# Supplementary material for: Differences in tail feather growth rate in storm-petrels breeding in the Northern and Southern hemisphere: a ptilochronological approach
Source: PeerJ. 2019 Oct 15;7:e7807. doi: 10.7717/peerj.7807 (PMC6798868; doi:10.7717/peerj.7807)
Supplement: Supplementary file 2 [file peerj-07-7807-s003.odt]

| Species | FGR (mm) | FL (mm) | FT | Source |
| --- | --- | --- | --- | --- |
| Chen caerulescens | 7.9 | 297 | P | Rohwer et al., 2009 |
| Chen rossii | 7.9 | 266 | P | Rohwer et al., 2009 |
| Anser anser | 5.5 | 308 | P | Rohwer et al., 2009 |
| Branta canadensis | 7.8 | 357 | P | Rohwer et al., 2009 |
| Branta leucopsis | 7.3 | 291 | P | Rohwer et al., 2009 |
| Branta bernicla | 5.7 | 258 | P | Rohwer et al., 2009 |
| Cygnus cygnus | 9 | 407 | P | Rohwer et al., 2009 |
| Cygnus olor | 6.9 | 406 | P | Rohwer et al., 2009 |
| Coscoroba coscoroba | 5 | 329 | P | Rohwer et al., 2009 |
| Anas platyrhynchos | 4.5 | 198 | P | Rohwer et al., 2009 |
| Gallus gallus | 4 | 192 | P | Rower et al., 2009 |
| Meleagris gallopavo | 7.5 | 395 | P | Rohwer et al., 2009 |
| Phasianus colchicus | 6.1 | 181 | P | Rohwer et al., 2009 |
| Oceanites oceanicus | 2.23 | 81.5 | R | This study |
| Fregetta tropica | 2.56 | 93.2 | R | This study |
| Phoebastria immutabilis | 2.8 | 232.7 | P | Langston & Rohwer, 1996 |
| Hydrobates pelagicus | 1.24 | 61.9 | R | This study |
| Oceanodroma leucorhoa | 1.42 | 91.5 | R | This study |
| Oceanodroma homochroa | 1.7 | 106 | P | Rohwer et al., 2009 |
| Grus leucogeranus | 9 | 450 | P | Rohwer et al., 2009 |
| Grus carunculatus | 11 | 475 | P | Rohwer et al., 2009 |
| Grus japonensis | 11 | 478 | P | Rohwer et al., 2009 |
| Grus grus | 9 | 438 | P | Rohwer et al., 2009 |
| Grus vipio | 9 | 428 | P | Rohwer et al., 2009 |
| Larus hyperboreus | 8 | 323 | P | Rohwer et al., 2009 |
| Larus marinus | 9.5 | 343 | P | Rohwer et al., 2009 |
| Falco tinnunculus | 4.2 | 194 | P | Rohwer et al., 2009 |
| Empidonax difficilis | 2.61 | 61.26 | R | de la Hera, DeSante, & Milá, 2012 |
| Empidonax occidentalis | 2.7 | 66.35 | R | de la Hera, DeSante, & Milá, 2012 |
| Empidonax fulvifrons | 2.41 | 56.56 | R | de la Hera, DeSante, & Milá, 2012 |
| Empidonax wrightii | 3.17 | 65.55 | R | de la Hera, DeSante, & Milá, 2012 |
| Empidonax oberholseri | 2.61 | 64.27 | R | de la Hera, DeSante, & Milá, 2012 |
| Empidonax virescens | 2.96 | 60.11 | R | de la Hera, DeSante, & Milá, 2012 |
| Contopus virens | 2.84 | 70.32 | R | de la Hera, DeSante, & Milá, 2012 |
| Contopus sordidulus | 2.69 | 71.37 | R | de la Hera, DeSante, & Milá, 2012 |
| Contopus cooperi | 2.97 | 77.83 | R | de la Hera, DeSante, & Milá, 2012 |
| Sayornis phoebe | 3.17 | 75.03 | R | de la Hera, DeSante, & Milá, 2012 |
| Myiarchus crinitus | 4.36 | 96.8 | R | de la Hera, DeSante, & Milá, 2012 |
| Vireo griseus | 2.47 | 50.16 | R | de la Hera, DeSante, & Milá, 2012 |
| Vireo huttoni | 2.22 | 54.85 | R | de la Hera, DeSante, & Milá, 2012 |
| Vireo olivaceus | 2.79 | 57.72 | R | de la Hera, DeSante, & Milá, 2012 |
| Lanius senator | 3.3 | 77 | P | Rohwer et al., 2009 |
| Lanius ludovicianus | 3.28 | 97.51 | R | Grubb & Yosef, 1994 |
| Lanius ludovicianus | 3.45 | 97.93 | R | Grubb & Yosef, 1994 |
| Lanius ludovicianus | 3.5 | 98.54 | R | Grubb & Yosef, 1994 |
| Lanius ludovicianus | 3.65 | 100.09 | R | Grubb & Yosef, 1994 |
| Corvus monedula | 3.6 | 178 | P | Rohwer et al., 2009 |
| Corvus frugilegus | 3.8 | 252 | P | Rohwer et al., 2009 |
| Pica pica | 5.18 | 156.5 | R | de la Hera et al., 2011 |
| Pica pica | 2.6 | 172 | P | Rohwer et al., 2009 |
| Garrulus glandarius | 5.44 | 148.8 | R | de la Hera et al., 2011 |
| Cyanocitta cristata | 4.52 | 112.66 | R | de la Hera, DeSante, & Milá, 2012 |
| Cyanocitta stelleri | 4.3 | 128.29 | R | de la Hera, DeSante, & Milá, 2012 |
| Perisoreus canadensis | 4.58 | 116.5 | R | Waite, 1990 |
| Perisoreus canadensis | 4.6 | 119.1 | R | Waite, 1990 |
| Baeolophus bicolor | 3.01 | 71.2 | R | de la Hera, DeSante, & Milá, 2012 |
| Baeolophus bicolor | 2.88 | 70.25 | R | Grubb & Cimprich, 1990 |
| Baeolophus bicolor | 2.94 | 71.9 | R | Grubb & Cimprich, 1990 |
| Baeolophus bicolor | 3.02 | 74.04 | R | Grubb & Cimprich, 1990 |
| Parus atricapillus | 2.68 | 59.92 | R | de la Hera, DeSante, & Milá, 2012 |
| Parus carolinensis | 2.34 | 52.84 | R | de la Hera, DeSante, & Milá, 2012 |
| Parus carolinensis | 2.18 | 52.51 | R | Grubb & Cimprich, 1990 |
| Parus carolinensis | 2.28 | 57.14 | R | Grubb & Cimprich, 1990 |
| Parus carolinensis | 2.29 | 54.99 | R | Grubb & Cimprich, 1990 |
| Parus major | 2.99 | 66.71 | R | de la Hera et al., 2011 |
| Parus major | 2.87 | 65.9 | R | Matysioková & Remeš, 2010 |
| Parus caeruleus | 2.43 | 53.86 | R | de la Hera et al., 2011 |
| Phylloscopus trochilus | 3.1 | 54 | P | Rohwer et al., 2009 |
| Phylloscopus collybita | 2.37 | 49.29 | R | de la Hera et al., 2011 |
| Psaltriparus minimus | 1.97 | 49.28 | R | de la Hera, DeSante, & Milá, 2012 |
| Aegithalos caudatus | 2.4 | 58.36 | R | de la Hera et al., 2011 |
| Acrocephalus scirpaceus | 2.66 | 54.91 | R | de la Hera et al., 2011 |
| Acrocephalus schoenobaenus | 2.38 | 48.28 | R | de la Hera et al., 2011 |
| Acrocephalus schoenobaenus | 2.6 | 53 | P | Rohwer et al., 2009 |
| Acrocephalus arundinaceus | 3.4 | 74 | P | Rohwer et al., 2009 |
| Sylvia atricapilla | 2.95 | 62.87 | R | Carbonell & Tellería,1999 |
| Sylvia atricapilla | 2.99 | 63.31 | R | Carbonell & Tellería,1999 |
| Sylvia atricapilla | 3.01 | 63.04 | R | Carbonell & Tellería,1999 |
| Sylvia atricapilla | 3.08 | 63.95 | R | de la Hera et al., 2011 |
| Sylvia borin | 3.04 | 59.04 | R | de la Hera et al., 2011 |
| Sylvia communis | 3.35 | 66.03 | R | de la Hera et al., 2011 |
| Prinia gracilis | 2.25 | 56.5 | R | Yosef, 1997 |
| Prinia gracilis | 2.33 | 57.2 | R | Yosef, 1997 |
| Toxostoma rufum | 4.13 | 109.81 | R | de la Hera, DeSante, & Milá, 2012 |
| Mimus polyglottos | 4.4 | 106.45 | R | de la Hera, DeSante, & Milá, 2012 |
| Dumetella carolinensis | 3.87 | 87.67 | R | de la Hera, DeSante, & Milá, 2012 |
| Sturnus vulgaris | 3.8 | 70 | R | White, Kennedy, & Stouffer, 1991 |
| Sturnus vulgaris | 3.8 | 69.4 | R | White, Kennedy, & Stouffer, 1991 |
| Sturnus vulgaris | 3.9 | 70.4 | R | White, Kennedy, & Stouffer, 1991 |
| Zoothera naevia | 3.6 | 95.3 | R | de la Hera, DeSante, & Milá, 2012 |
| Hylocichla mustelina | 3.41 | 78.37 | R | de la Hera, DeSante, & Milá, 2012 |
| Catharus ustulatus | 3.59 | 75.67 | R | de la Hera, DeSante, & Milá, 2012 |
| Catharus guttatus | 3.51 | 74.49 | R | de la Hera, DeSante, & Milá, 2012 |
| Catharus fuscescens | 3.64 | 76.31 | R | de la Hera, DeSante, & Milá, 2012 |
| Catharus minimus | 3.86 | 81.59 | R | de la Hera, DeSante, & Milá, 2012 |
| Turdus migratorius | 4.44 | 105.84 | R | de la Hera, DeSante, & Milá, 2012 |
| Turdus merula | 4.6 | 116 | R | de la Hera et al., 2011 |
| Turdus philomelos | 4.07 | 88.31 | R | de la Hera et al., 2011 |
| Sialia sialis | 3.13 | 70.34 | R | de la Hera, DeSante, & Milá, 2012 |
| Sialia mexicana | 3.15 | 74.84 | R | de la Hera, DeSante, & Milá, 2012 |
| Luscinia megarhynchos | 3.35 | 69.42 | R | de la Hera et al., 2011 |
| Luscinia luscinia | 4.2 | 69 | P | Rohwer et al., 2009 |
| Luscinia svecica | 3.2 | 57 | P | Rohwer et al., 2009 |
| Oenanthe oenanthe | 3.8 | 72 | P | Rohwer et al., 2009 |
| Phoenicurus phoenicurus | 3.04 | 62.38 | R | de la Hera et al., 2011 |
| Erithacus rubecula | 2.56 | 61.98 | R | de la Hera et al., 2011 |
| Sitta carolinensis | 2.51 | 52.36 | R | de la Hera, DeSante, & Milá, 2012 |
| Sitta carolinensis | 1.98 | 51.32 | R | Dolby & Grubb, 1998 |
| Sitta carolinensis | 2.37 | 51.73 | R | Grubb & Cimprich, 1990 |
| Bombycilla cedrorum | 3.06 | 63.53 | R | de la Hera, DeSante, & Milá, 2012 |
| Regulus calendula | 2.3 | 49.14 | R | de la Hera, DeSante, & Milá, 2012 |
| Carduelis carduelis | 2.69 | 51.22 | R | de la Hera et al., 2011 |
| Carduelis tristis | 2.55 | 53.26 | R | de la Hera, DeSante, & Milá, 2012 |
| Carduelis psaltria | 2.34 | 45.99 | R | de la Hera, DeSante, & Milá, 2012 |
| Carduelis pinus | 2.5 | 51.37 | R | de la Hera, DeSante, & Milá, 2012 |
| Carduelis flammea | 2.9 | 58 | P | Rohwer et al., 2009 |
| Carduelis chloris | 2.73 | 57.45 | R | de la Hera et al., 2011 |
| Carduelis chloris | 2.4 | 70 | P | Rohwer et al., 2009 |
| Carpodacus purpureus | 3 | 64.21 | R | de la Hera, DeSante, & Milá, 2012 |
| Carpodacus cassinii | 2.99 | 67.83 | R | de la Hera, DeSante, & Milá, 2012 |
| Carpodacus mexicanus | 3.01 | 66.99 | R | de la Hera, DeSante, & Milá, 2012 |
| Carpodacus mexicanus | 3 | 64 | P | Rohwer et al., 2009 |
| Pyrrhula pyrrhula | 2.4 | 73 | P | Rohwer et al., 2009 |
| Coccothraustes vespertinus | 3.16 | 69.71 | R | de la Hera, DeSante, & Milá, 2012 |
| Fringilla coelebs | 3.42 | 70.85 | R | de la Hera et al., 2011 |
| Miliaria calandra | 3.59 | 81.69 | R | de la Hera et al., 2011 |
| Amphispiza bilineata | 3.26 | 62.4 | R | de la Hera, DeSante, & Milá, 2012 |
| Spizella breweri | 3.05 | 62.53 | R | de la Hera, DeSante, & Milá, 2012 |
| Spizella pusilla | 3.01 | 66.08 | R | de la Hera, DeSante, & Milá, 2012 |
| Spizella passerina | 3 | 63.1 | R | de la Hera, DeSante, & Milá, 2012 |
| Passerella iliaca | 3.6 | 77.78 | R | de la Hera, DeSante, & Milá, 2012 |
| Spizella arborea | 2.83 | 68.48 | R | White & Kennedy, 1992 |
| Junco hyemalis | 3.09 | 66.84 | R | de la Hera, DeSante, & Milá, 2012 |
| Junco phaeonotus | 3.07 | 72.89 | R | de la Hera, DeSante, & Milá, 2012 |
| Zonotrichia atricapilla | 3.58 | 79.69 | R | de la Hera, DeSante, & Milá, 2012 |
| Zonotrichia leucophrys | 3.27 | 73.04 | R | de la Hera, DeSante, & Milá, 2012 |
| Zonotrichia albicollis | 3.33 | 76 | R | de la Hera, DeSante, & Milá, 2012 |
| Pipilo erythrophthalmus | 3.65 | 88.91 | R | de la Hera, DeSante, & Milá, 2012 |
| Pipilo fuscus | 3.68 | 92.36 | R | de la Hera, DeSante, & Milá, 2012 |
| Pipilo crissalis | 3.7 | 94.58 | R | de la Hera, DeSante, & Milá, 2012 |
| Melospiza lincolnii | 2.52 | 56.15 | R | de la Hera, DeSante, & Milá, 2012 |
| Melospiza georgiana | 2.73 | 58.79 | R | de la Hera, DeSante, & Milá, 2012 |
| Melospiza melodia | 2.95 | 63.62 | R | de la Hera, DeSante, & Milá, 2012 |
| Icterus galbula | 3.51 | 71.29 | R | de la Hera, DeSante, & Milá, 2012 |
| Icterus bullockii | 3.59 | 78.81 | R | de la Hera, DeSante, & Milá, 2012 |
| Molothrus ater | 3.79 | 77.4 | R | de la Hera, DeSante, & Milá, 2012 |
| Agelaius phoeniceus | 4.14 | 86.34 | R | de la Hera, DeSante, & Milá, 2012 |
| Seiurus aurocapilla | 2.89 | 58.3 | R | de la Hera, DeSante, & Milá, 2012 |
| Vermivora celata | 2.57 | 51.41 | R | de la Hera, DeSante, & Milá, 2012 |
| Vermivora virginiae | 2.59 | 51.66 | R | de la Hera, DeSante, & Milá, 2012 |
| Vermivora pinus | 2.55 | 47.21 | R | de la Hera, DeSante, & Milá, 2012 |
| Mniotilta varia | 2.77 | 53.37 | R | de la Hera, DeSante, & Milá, 2012 |
| Seiurus motacilla | 2.9 | 56.17 | R | de la Hera, DeSante, & Milá, 2012 |
| Seiurus noveboracensis | 3.07 | 57.37 | R | de la Hera, DeSante, & Milá, 2012 |
| Protonotaria citrea | 2.62 | 47.67 | R | de la Hera, DeSante, & Milá, 2012 |
| Limnothlypis swainsonii | 2.58 | 52.26 | R | de la Hera, DeSante, & Milá, 2012 |
| Oporornis formosus | 2.59 | 51.12 | R | de la Hera, DeSante, & Milá, 2012 |
| Oporornis tolmiei | 2.62 | 57.33 | R | de la Hera, DeSante, & Milá, 2012 |
| Oporornis philadelphia | 2.47 | 49.08 | R | de la Hera, DeSante, & Milá, 2012 |
| Geothlypis trichas | 2.49 | 50.74 | R | de la Hera, DeSante, & Milá, 2012 |
| Dendroica pensylvanica | 2.62 | 49.75 | R | de la Hera, DeSante, & Milá, 2012 |
| Dendroica petechia | 2.62 | 49.23 | R | de la Hera, DeSante, & Milá, 2012 |
| Dendroica pinus | 2.73 | 58.87 | R | de la Hera, DeSante, & Milá, 2012 |
| Parula americana | 2.24 | 42.34 | R | de la Hera, DeSante, & Milá, 2012 |
| Dendroica magnolia | 2.56 | 52.12 | R | de la Hera, DeSante, & Milá, 2012 |
| Wilsonia citrina | 2.7 | 56.63 | R | de la Hera, DeSante, & Milá, 2012 |
| Wilsonia pusilla | 2.58 | 51.03 | R | de la Hera, DeSante, & Milá, 2012 |
| Ergaticus ruber | 4.02 | 98.58 | R | de la Hera, DeSante, & Milá, 2012 |
| Wilsonia canadensis | 2.71 | 53.35 | R | de la Hera, DeSante, & Milá, 2012 |
| Helmitheros vermivorum | 2.72 | 52.08 | R | de la Hera, DeSante, & Milá, 2012 |
| Icteria virens | 3.73 | 76.69 | R | de la Hera, DeSante, & Milá, 2012 |
| Passerina caerulea | 3.55 | 71.62 | R | de la Hera, DeSante, & Milá, 2012 |
| Passerina amoena | 3.23 | 59.73 | R | de la Hera, DeSante, & Milá, 2012 |
| Passerina cyanea | 2.91 | 55.04 | R | de la Hera, DeSante, & Milá, 2012 |
| Passerina ciris | 3.13 | 60.84 | R | de la Hera, DeSante, & Milá, 2012 |
| Piranga rubra | 3.83 | 79.68 | R | de la Hera, DeSante, & Milá, 2012 |
| Piranga olivacea | 3.58 | 72.82 | R | de la Hera, DeSante, & Milá, 2012 |
| Piranga ludoviciana | 3.58 | 77.42 | R | de la Hera, DeSante, & Milá, 2012 |
| Piranga flava | 3.24 | 84.42 | R | de la Hera, DeSante, & Milá, 2012 |
| Pheucticus melanocephalus | 3.47 | 82.03 | R | de la Hera, DeSante, & Milá, 2012 |
| Pheucticus ludovicianus | 3.68 | 77.59 | R | de la Hera, DeSante, & Milá, 2012 |
| Sporophila torqueola | 2.33 | 44.87 | R | de la Hera, DeSante, & Milá, 2012 |
| Motacilla alba | 4.5 | 67 | P | Rohwer et al., 2009 |
| Passer domesticus | 3.17 | 64.51 | R | de la Hera et al., 2011 |
| Passer domesticus | 3.03 | 62.93 | R | de la Hera, DeSante, & Milá, 2012 |
| Passer domesticus | 2.7 | 57 | P | Rohwer et al., 2009 |
| Passer montanus | 3.07 | 57.68 | R | de la Hera et al., 2011 |
| Pandion haliaetus | 7.9 | 372 | P | Rohwer et al., 2009 |
| Gypaetus barbatus | 6.6 | 600 | P | Rohwer et al., 2009 |
| Gyps africanus | 4.4 | 440 | P | Rohwer et al., 2009 |
| Picoides pubescens | 2.39 | 50.04 | R | Grubb & Cimprich, 1990 |
| Picoides pubescens | 2.45 | 49.71 | R | Grubb & Cimprich, 1990 |
| Picoides pubescens | 2.34 | 50.07 | R | Grubb, 1989 |
| Picoides pubescens | 2.35 | 50.16 | R | Grubb, 1989 |
| Picoides pubescens | 2.48 | 52.64 | R | Grubb, 1989 |
| Picoides pubescens | 2.6 | 50.74 | R | Grubb, 1989 |
| Halcyon leucocephala | 3.6 | 78 | P | Rohwer et al., 2009 |
| Streptopelia roseogrisea | 5.5 | 129 | P | Rohwer et al., 2009 |

References

Carbonell, R., & Tellería, J. L. (1999). Feather traits and ptilochronology as indicators of stress in Iberian blackcaps Sylvia atricapilla. Bird Study, 46(2), 243–248. https://doi.org/10.1080/00063659909461136

de la Hera, I., DeSante, D. F., & Milá, B. (2012). Feather growth rate and mass in Nearctic passerines with variable migratory behavior and molt pattern. The Auk, 129(2), 222–230. https://doi.org/10.1525/auk.2012.11212

de la Hera, I., Schaper, S. V, Díaz, J. A., Pérez-Tris, J., Bensch, S., & Tellería, J. L. (2011). How much variation in the molt duration of passerines can be explained by the growth rate of tail feathers? The Auk, 128(2), 321–329. https://doi.org/10.1525/auk.2011.10181

Dolby, A. S., & Grubb, T. C. (1998). Benefits to satellite members in mixed-species foraging groups: An experimental analysis. Animal Behaviour, 56(2), 501–509. https://doi.org/10.1006/anbe.1998.0808

Grubb, T. C. (1989). Ptilochronology - feather growth bars as indicators of nutritional-status. The Auk, 106(2), 314–320.

 Grubb, T. C., & Cimprich, D. A. (1990). Supplementary food improves the nutritional condition of wintering woodland birds: evidence from ptilochronology. Ornis Scandinavica, 21(4), 277–281.

Grubb, T. C., & Yosef, R. (1994). Habitat-specific nutritional condition in loggerhead shrikes (Lanius ludovicianus): Evidence from ptilochronology. The Auk, 111(3), 756–759. 

Langston, N. E., & Rohwer, S. (1996). Molt-breeding trade-offs in albatrosses: life history implications for big birds. Oikos, 76(3), 498–510.

Matysioková, B., & Remeš, V. (2010). Assessing the usefulness of ptilochronology in the study of melanin- and carotenoid-based ornaments in the great tit Parus major. Ibis, 152(2), 397–401. https://doi.org/10.1111/j.1474-919X.2009.01002.x

Rohwer, S., Ricklefs, R. E., Rohwer, V. G., & Copple, M. M. (2009). Allometry of the duration of flight feather molt in birds. PLoS Biology, 7(6). [https://doi.org/10.1371/journal.pbio.1000132](https://doi.org/10.1371/journal.pbio.1000132)

Saino, N., Romano, M., Caprioli, M., Ambrosini, R., Rubolini, D., Scandolara, C. and Romano, A. (2012). A ptilochronological study of carry-over effects of conditions during wintering on breeding performance in the barn swallow Hirundo rustica. Journal of Avian Biology 43, 513–524 

Waite, T. A. (1990). Effects of caching supplemental food on induced feather regeneration in wintering gray jays Perisoreus canadensis: a ptilochronology study. Ornis Scandinavica, 21(2), 122–128. https://doi.org/10.2307/3676807

White, D. W., & Kennedy, E. D. (1992). Growth of induced feathers in photostimulated American tree sparrows. The Condor, 94(2), 543–545. https://doi.org/10.2307/1369230

White, D. W., Kennedy, E. D., & Stouffer, P. C. (1991). Feather regrowth in female European starlings rearing broods of different sizes. The Auk, 108(4), 889–895. https://doi.org/10.1016/j.ress.2016.07.018

Yosef, R. (1997). On habitat-specific nutritional condition in graceful warblers Prinia gracilis: Evidence from ptilochronology. Journal für Ornithologie, 138(3), 309–313. https://doi.org/10.1007/BF01651556
