# Supplementary material for: Differences in tail feather growth rate in storm-petrels breeding in the Northern and Southern hemisphere: a ptilochronological approach
Source: PeerJ. 2019 Oct 15;7:e7807. doi: 10.7717/peerj.7807 (PMC6798868; doi:10.7717/peerj.7807)
Supplement: Supplementary file 3 [file peerj-07-7807-s004.odt]

| Feather type | Predictor | Pagel’s λ | Value | SE | t-value | Pr(>│t│) |
| --- | --- | --- | --- | --- | --- | --- |
| Rectrices | Intercept | 0.987 | -0.828 | 0.080 | -10.345 | < 0.001 |
| Log10 FL | 0.658 | 0.040 | 16.576 | < 0.001 |
| Primaries | Intercept | 0.903 | -0.276 | 0.241 | -1.145 | 0.259 |
| Log10 FL | 0.419 | 0.100 | 4.187 | < 0.001 |
